# A multicenter study of genetic testing for Parkinson’s disease in the clinical setting

**DOI:** 10.1038/s41531-022-00408-6

**Published:** 2022-11-04

**Authors:** Anja Kovanda, Valentino Rački, Gaber Bergant, Dejan Georgiev, Dušan Flisar, Eliša Papić, Marija Brankovic, Milena Jankovic, Marina Svetel, Nataša Teran, Aleš Maver, Vladimir S. Kostic, Ivana Novakovic, Zvezdan Pirtošek, Martin Rakuša, Vladimira Vuletić, Borut Peterlin

**Affiliations:** 1 Clinical Institute of Genomic Medicine, University Medical Centre Ljubljana, Ljubljana, Slovenia; 2 Department of Neurology, University of Rijeka, Faculty of Medicine, Rijeka, Croatia; 3 Department of Neurology, University Medical Centre Ljubljana, Ljubljana, Slovenia; 4 Artificial Intelligence Lab, Faculty of Computer and Information Sciences, University of Ljubljana, Ljubljana, Slovenia; 5 Neurology Clinic, Faculty of Medicine, University of Belgrade, Belgrade, Serbia; 6 Neurology Clinic, UCCS, Belgrade, Serbia; 7 Institute of Human Genetics and Neurology Clinic, Faculty of Medicine, University of Belgrade, Belgrade, Serbia; 8 Department of Neurology, Faculty of Medicine, University of Ljubljana, Ljubljana, Slovenia; 9 Department of Neurology, University Medical Centre Maribor, Maribor, Slovenia

**Keywords:** Parkinson's disease, Genetic testing

## Abstract

Parkinson’s disease (PD) guidelines lack clear criteria for genetic evaluation. We assessed the yield and rationale of genetic testing for PD in a routine clinical setting on a multicenter cohort of 149 early-onset and familial patients by exome sequencing and semi-quantitative multiplex ligation-dependent probe amplification of evidence-based PD-associated gene panel. We show that genetic testing for PD should be considered for both early-onset and familial patients alike, and a clinical yield of about 10% in the Caucasian population can be expected.

Parkinson’s disease (PD) is a common extrapyramidal disorder with an onset around 65 years of age. Onset before age 50 is considered early-onset PD (EOPD), and ~15% of PD patients are familial (FPD). PD is a multifactorial disease, and an estimated 5–10% can be contributed to monogenic causes. Whereas patients with EOPD and FPD are generally considered to be at increased risk for monogenic genetic predisposition there is limited evidence on the clinical use of comprehensive genetic testing in these populations. Predominant pathogenic and likely pathogenic variants (P/LP) in PD genes are population and phenotype specific, and identifying those patients remains a diagnostic challenge^1^. Additionally, copy-number variants (CNV) may represent potential missing heritability^2^, and their presence should be examined in genes where known P/LP CNVs have been identified, such as *SNCA*, as well as in patients showing heterozygous SNV variants in AR PD genes such as *PARK2* and *PINK*^3^.

The latest recommendations by EFNS/MDS-ES from 2013 regarding PD genetic testing precede accessibility of next-generation sequencing (NGS) in routine diagnostics and suggest testing should be individually decided and performed only for a few select genes^4^. In the last decade availability of NGS technology has enabled research of PD genetics to expand to many potential genes as well as rare variants^5–9^. However, the selection of the target gene panel is critical for effective translation into clinical practice, as clear clinical validity of an evaluated gene is required to classify variants according to the ACMG criteria for diagnosing monogenic disorders^10^. Furthermore, clinical reporting of variants must consider the proposed inheritance model for each PD-gene^11^.

The majority of previous genetic studies of PD looked at many target genes and reported several rare variants and candidate PD genes, with yields varying from 7.5% to as much as 43.5%^1,5–9,12–15^. The higher reported yields should be examined carefully for clinical validity, as many of these genes do not satisfy the stringency of ACMG criteria for reporting, with clinically reportable results being limited to evidence-based PD-associated genes only. Indeed, to the best of our knowledge, there is only one study that focused on the causality of detected variants, like our own study, and reported a 7.5% clinical yield^9^. This highlights the difference between actual reportable variants compared to other studies that reported variants based on their P/LP prediction alone.

This lack of clear clinical guidelines on which patients warrant testing, and no consensus on the exact genes to test for PD, currently translates to a lack of genetic testing in routine clinical practice. Indeed, a survey from 2019 reported that 41% of experienced PD physicians did not perform genetic testing in the prior year and more than 80% referred less than 11 patients in that period^16^. Given the relatively high cost and low yield of genetic testing in PD, clinicians need to have clear data on which they can base their testing decisions.

Therefore, we aimed to evaluate the yield and rationale of genetic testing for PD in the routine clinical setting by testing EOPD and FPD patients, by using exome sequencing (ES) of a 35 evidence-based PD-associated gene panel, as evaluated by expert groups ClinGen^17^ and Genomics England^18^, followed by the complementary multiplex ligation-dependent probe amplification (MLPA) method. We hypothesize that routine genetic testing of PD should include only clearly disease-associated genes, and only variants consistent with the inheritance model of each gene are clinically relevant and represent true yield.

A total of 149 EOPD and FPD patients of Slavic ethnicity were included in the study (Table 1). Using ES, we detected a genetic contributor in 15 patients (10.1%), of which 14 had P/LP variants in *GBA*, and one had two P/LP variants in *PARK2*, an SNV and an exon 5 deletion detected using MLPA (Tables 1 and 2). No difference in the yield of causative P/LP variants was observed between the EOPD and FPD groups (*X*^2^ (1, *N* = 15) = 0.04, *p* = 0.85) (Table 1).

**Table 1:** Patient’s characteristics.

Patients	*N* (%)	Sex M/F (%/%)	Average age-of-onset years (range)	Causative variant *N* (%)	No causative variant *N* (%)
All	149 (100.0)	92/57 (61.7/38.3)	47 (24–87)	15 (10.1)	134 (89.9)
EOPD	76 (51.0)	51/25 (67.1/32.9)	42 (24–50)	8 (10.5)	68 (89.5)
FPD	73 (49.0)	41/32 (56.2/43.8)	52 (25–87)	7 (9.6)	66 (90.4)

*EOPD* sporadic early-onset PD, *FPD* familial PD.

**Table 2:** Causative pathogenic and likely pathogenic variants.

Patient	Age of onset (years)	Patient group	Gene variant	Class	Applied ACMG criteria^a^
P006	41	EOPD	*GBA NM_000157.4:c.1226A* > *G*	P	PS3, PM2, PM3_VSTR, PP2
P012	57	FPD			
P014	68				
P015	69				
P002	32	EOPD	*GBA NM_000157.4:c.1448T* > *C*	P	PS3, PM2, PM3_VSTR, PP2
P003	46				
P013	59	FPD			
P007	43	EOPD	*GBA NM_000157.4:c.586A* > *C*	LP	PS3, PM2, PM3, PP2
P001	49				
P011	51	FPD			
P005	36	EOPD	*GBA NM_000157.4:c.1289C* > *T*	P	PS3, PM2, PM3_STR, PP2
P004	50				
P010	50	FPD	*GBA NM_000157.4:c.1090G* > *A*	P	PS3_MOD, PM2, PM3_STR, PM5_SUP, PP2
P009	36	FPD	*GBA NM_000157.4:c.115* + *1G* > *A*	P	PVS1, PM2, PM3_VSTR
P008	34	EOPD	*PARK2 NM_004562.3:c.823C* > *T*	P	PS3, PM2, PM3_VSTR, PP2
			*PARK2 Exon 5 deletion*	LP	PVS1_MOD, PM3_STR, PM2

*P* pathogenic, *LP* likely pathogenic, *EOPD* sporadic early-onset PD, *FPD* familial PD,

^a^ACMG criteria modifiers = very strong (VSTR), strong (STR), moderate (MOD), or supporting (SUP).

The involvement of *GBA* P/LP variants in familial PD and their penetrance is complex^19–22^, and our results showing P/LP variants in *GBA* to be the main reportable findings in our PD patients are in line with previous studies of PD patients of European ancestry^5,7^ as well as international studies^23^.

We identified the majority of causative P/LP using ES, however, MLPA revealed an additional 3 CNV in *PARK2* (Supplementary information), one of which revealed a compound heterozygosity, leading to the genetic diagnosis in the patient. Therefore, to comprehensively address molecular pathology in the PD-associated genes, ES should be followed by MLPA as a complementary method. The two additionally detected heterozygous P/LP copy-number variants (CNV) in *PARK2* detected by MLPA were classified as low-risk variants or risk-factors with incomplete penetrance, as no additional SNV P/LP in *PARK2* were found in these two patients (Supplementary information).

Finally, variants of uncertain significance (VUS) in PD-associated genes were identified in 22 patients using ES (*GBA* in 8, *LRRK2* in 5, *ATP13A2* in 2, and *ATP1A3, CSF1R, FTL, PLA2G6, SNCA*, *TUBB4A, and VPS35* each in one patient) (Supplementary information). At the moment, these results do not constitute clinically reportable findings, but since their role in PD pathogenesis may be resolved in the future, annual re-interpretation of such findings is advisable.

To conclude, our study identifies that genetic testing for PD should be considered in EOPD and FPD patients alike. Furthermore, a clear clinical testing focus should remain on a comprehensive set of validated/curated genes, and there is currently no rationale to test and classify variants in other genes in the clinical setting. By using these recommendations, a clinical yield of about 10% in the Caucasian population can be expected using ES and MLPA combined.

## Methods

Our study cohort included 149 patients with PD, consecutively referred for routine genetic testing at the Clinical Institute of Genomic Medicine (CIGM)(Slovenia), and patients from Neurology departments Rijeka (Croatia) and Belgrade (Serbia), from January 2014 to October 2021.

All medical procedures in the study were performed in accordance with the ethical standards of the 1964 Helsinki Declaration and its later amendments or comparable ethical standards and national regulations of Slovenia, Croatia and Serbia.Written informed consent for genetic testing was obtained from the patients during their clinical appointment in the Slovenian, Croatian or Serbian language, granting the Clinical Institute of Genomic Medicine, where all the genetic testing was performed, rights to publish the findings of genetic testing in de-identified form in scientific literature. The informed consent statement was prepared according to National review board guidelines and approved by the Institutional Ethics Board at the University Medical Centre Ljubljana, Slovenia, and subsequently translated and approved for use by the Institutional Boards of Faculty of Medicine Rijeka, Croatia, and Faculty of Medicine, Belgrade, Serbia.

The inclusion criteria were confirmed clinical PD diagnosis based on United Kingdom Parkinson’s Disease Society Brain Bank clinical diagnostic criteria^24^, and either sporadic EOPD (<50 years) or familial PD (FPD) with at least one affected 1st-degree relative. Exclusion criteria were inconsistent clinical presentation, Parkinson-plus syndromes, idiopathic sporadic late-onset PD, or insufficient clinical information.

### Gene panel analysis

ES was performed at CIGM using standardized protocols in use at the time of processing. In brief, we performed ES capture using TruSight One, TruSight Exome, Nextera Coding Exome, and IDT Exome capture kits (Illumina, San Diego, CA), Agilent SureSelect Human All Exon v2, v5, v6, and v7 capture kits (Agilent Technologies, Santa Clara, CA), as well as Twist Library Preparation Kit (Twist Bioscience, San Francisco, CA). Sequencing was performed on Illumina sequencing platforms in either 2 × 100 or 2 × 150 paired-end sequencing mode.

Initially, sequencing data analysis and variant interpretation were performed as previously described^25,26^. Archived raw data from all samples were re-analyzed using the most current software and annotation databases using a gene panel with ClinGen validated PD-associated genes^17^ (https://clinicalgenome.org/affiliation/40079/) and Genomics England panel for Parkinson’s disease and complex parkinsonism Version 1.7^18^ (https://panelapp.genomicsengland.co.uk/panels/39/). Genes where tandem repeat expansions were previously reported as pathogenic have not been analyzed due to NGS limitations. Our final panel included the following genes (genes included in both panels are indicated in bold): *ATP13A2*, *ATP1A3*, *C19orf12*, *CSF1R*, *DCTN1*, *DNAJC6*, *FBXO7*, *FTL*, ***GBA***, *GCH1*, *GRN*, ***LRRK2***, *LYST*, *MAPT*, *OPA3*, *PANK2*, ***PARK7***, ***PINK1***, *PLA2G6*, ***PARK2***, *PRKRA*, *PTRHD1*, *RAB39B*, *SLC30A10*, *SLC39A14*, *SLC6A3*, ***SNCA***, *SPG11*, *SPR*, *SYNJ1*, *TH*, *TUBB4A*, *VPS13A*, ***VPS35***, and *WDR45*.

Identified variants were classified according to the ACMG and AMP 2015 joint consensus recommendation^10^. The evidence support level was additionally weighted according to the ACGS recommendations where applicable^27^. Pathogenic and likely pathogenic (P/LP) variants in patients consistent with the inheritance model of individual gene disorders were considered clinically relevant.

### Multiplex ligation-dependent probe amplification analysis

The semi-quantitative Multiplex Ligation-dependent Probe Amplification (MLPA) SALSA MLPA Probemixes P051 and P052 Parkinson mix assay (MRC Holland, Amsterdam, The Netherlands) were used for the detection of deletions or duplications in *SNCA*, *PARK2*, *UCHL1*, *PINK1*, *PARK7*, *ATP13A2*, *LRRK2*, and *GCH1* genes.

## Supplementary information

Supplementary table 1

## Data Availability

The authors declare that the data supporting the findings of this study are available within the paper and its supplementary information files. Reported P/LP variants’ ClinVar database accession numbers (https://www.ncbi.nlm.nih.gov/clinvar/)^28^ are available in Supplementary information. The raw ES datasets generated during and analyzed during the current study are available from the corresponding author B.P. on reasonable request.
