# Supplementary material for: A multicenter study of genetic testing for Parkinson’s disease in the clinical setting
Source: NPJ Parkinsons Dis. 2022 Nov 4;8:149. doi: 10.1038/s41531-022-00408-6 (PMC9636217; doi:10.1038/s41531-022-00408-6)
Supplement: Supplementary file 1 — Supplementary table 1 [file 41531_2022_408_MOESM1_ESM.docx]

**Supplementary table 1:** Discovered variants in the PD-gene panel.

| Gene (transcript) / Inheritance | Variant | Variant  class | N total | N EOPD | N  FPD | Patient ID | ClinVar  variant ID |
| --- | --- | --- | --- | --- | --- | --- | --- |
| *GBA* (NM_000157.4) / AD | **c.1226A>G** | **P** | **4** | **1** | **3** | **P006, P012, P014, P015** | **4290** |
|  | **c.1448T>C** | **P** | **3** | **2** | **1** | **P002, P003, P013** | **4288** |
|  | **c.586A>C** | **LP** | **3** | **2** | **1** | **P001, P007, P011** | **4300** |
|  | **c.1289C>T** | **P** | **2** | **2** | **0** | **P004, P005** | **931820** |
|  | **c.1090G>A** | **P** | **1** | **0** | **1** | **P010** | **4318** |
|  | **c.115+1G>A** | **P** | **1** | **0** | **1** | **P009** | **93445** |
|  | c.1093G>A | VUS | 3 | 1 | 2 | P027, P035, P041 | 199044 |
|  | c.247C>T | VUS | 1 | 0 | 1 | P113 | 806230 |
|  | c.428T>G | VUS | 1 | 1 | 0 | P026 | / |
|  | c.1279G>A | VUS | 1 | 0 | 1 | P034 | 493050 |
|  | c.1389-33delC | VUS | 1 | 0 | 1 | P040 | 930901 |
|  | c.1579T>A | VUS | 1 | 1 | 0 | P028 | / |
| *PARK2* (NM_004562.3) / AR | **c.823C>T** | **P** | **1** | **1** | **0** | **P008** | **7050** |
|  | **Exon 5 deletion** | **LP** | **1** | **1** | **0** |  | **417464** |
|  | Exon 3 and 4 deletion | P | 1 | 1 | 0 | P016 | 650192 |
|  | Exon 4 duplication | LP | 1 | 0 | 1 | P019 | 417461 |
| *LRRK2* (NM_198578.4) / AD | c.1163T>C | VUS | 1 | 1 | 0 | P030 | / |
|  | c.2576G>T | VUS | 1 | 0 | 1 | P044 | / |
|  | c.3223G>A | VUS | 1 | 1 | 0 | P029 | / |
|  | c.5173C>T | VUS | 1 | 0 | 1 | P037 | 39204 |
|  | c.7475G>T | VUS | 1 | 0 | 1 | P036 | 931900 |
| *ATP13A2* (LRG_834t1) / AR | c.58A>C | VUS | 1 | 1 | 0 | P023 | / |
|  | c.3490C>A | VUS |  |  |  |  | 788230 |
|  | c.1759G>A | VUS | 1 | 0 | 1 | P038 | / |
|  | *37T>C | VUS |  |  |  |  | / |
| *PLA2G6* (LRG_1015t1) / AR | c.1111G>A | LP | 1 | 1 | 0 | P020 | 985636 |
|  | c.343C>T | VUS | 1 | 1 | 0 |  | 805204 |
| *ATP1A3* (NM_001256214.1) / AD | c.163T>C | VUS | 1 | 1 | 1 | P033 | 931868 |
| *CSF1R* (NM_001288705.3) / AR/AD | c.647G>A | VUS | 1 | 1 | 1 | P039 | 374038 |
| *FTL* (NM_000146.4) / AD | c.515_516delTC | VUS | 1 | 1 | 0 | P025 | / |
| *SNCA* (NM_000345.3) /AD | c.44T>C | VUS | 1 | 1 | 1 | P045 | 871101 |
| *TUBB4A* (NM_001289123.1) / AD | c.1073_1089del | VUS | 1 | 1 | 0 | P032 | / |
| *VPS35* (NM_018206.6) / AD | c.2210C>T | VUS | 1 | 1 | 1 | P043 | 487677 |

AD = autosomal dominant, AR = autosomal recessive, P = pathogenic, LP = likely pathogenic, VUS = variant of unknown significance. Variants reported as causative in patients are indicated in **bold**.
